# A population-based study of interactions between high-risk human papillomavirus infection and vaginal local cytokines CD4 CD8 IL-10 with cervical intraepithelial neoplasia

**DOI:** 10.3389/fonc.2025.1634489

**Published:** 2025-10-10

**Authors:** Ruoxi Zhu, Aimin Yang, Wenhao Wang, Weihong Zhao, Wei Wang, Zhilian Wang, Jintao Wang, Yongli Hou, Xiaoqiang Su, Lili Zhang, Bo Feng, Jing Yang, Zhe Wang, Xiaofen Niu, Weiguo Lv, Zhican Qu, Min Hao

**Affiliations:** ^1^ Departments of Obstetrics and Gynecology, Second Hospital of Shanxi Medical University, Taiyuan, Shanxi, China; ^2^ Department of Medicine and Therapeutics, The Chinese University of Hong Kong, Prince of Wales Hospital, Hong Kong, Hong Kong SAR, China; ^3^ Hong Kong Institute of Diabetes and Obesity, The Chinese University of Hong Kong, Hong Kong, Hong Kong SAR, China; ^4^ Department of Epidemiology, School of Public Health, Shanxi Medical University, Taiyuan, Shanxi, China; ^5^ Department of Gynecologic Oncology, Women’s Hospital, School of Medicine, Zhejiang University, Hangzhou, Zhejiang, China

**Keywords:** cervical intraepithelial neoplasia, cytokines, CD4, CD8, IL-10, high-risk human papillomavirus infection, cervical cancer

## Abstract

**Background:**

Cluster of Differentiation-4(CD4),Cluster of Differentiation-8(CD8), and interleukin-10 (IL-10) have long been considered to be related to cervical cancer, but the exact relationship remains unclear. Few studies investigated the relationship between CD4,CD8,IL-10, and high-risk human papillomavirus (HPV) with risk of cervical intraepithelial neoplasia (CIN).

**Objective:**

Our aim is to evaluate the relationship between CD4, CD8, IL-10, and high-risk HPV infection with the risk of CIN, as well as their interactions on CIN.

**Design:**

In 2014-2015, a cross-sectional study of screening data was conducted among 2285 women aged 19–65 years who participated in an ongoing community-based cohort of 40,000 women in Shanxi, China. Using categorical and spline analyses to evaluate the relationship between local vaginal fluids of CD4,CD8,CD4/CD8,IL-10, and CIN risk. A total of 1,503 controls were followed up until January 31, 2019. A nested case-control study was used to assess the relationship between vaginal lavage CD4, CD8, CD4/CD8, and IL-10 levels and the risk of CIN progression.

**Results:**

After adjusting for possible confounding factors,CD4 and CD8 levels were positively related to CIN risk (the 1st versus 4th quartile CD4,CD8 OR = 0.45[0.34, 0.60] and 0.34[0.26, 0.45] for CIN1, 0.32 [0.21, 0.48] and 0.24 [0.16, 0.38] for CIN2/3). Increased CD4 and CD8 levels were positively related to the occurrence of CIN(P-overall<0.01).CD4/CD8 levels and the risk of CIN1 followed a nonlinear “U-shape” (P-nonlinear <0.01). IL-10 levels and the risk of CIN1 followed a nonlinear “n-shape”(P-nonlinear <0.01).IL-10 levels were inversely related to the occurrence of CIN2/3(OR = 3.87, [2.49, 6.00],P-overall<0.01). The highest risk of CIN was observed in women with high-risk HPV, whose CD4 and CD8 levels were the highest(P-interaction < 0.01).Patients with the lowest IL-10 levels(IL-10 ≤ 53.17pg/ml) who are positive for high-risk HPV infection have the highest risk of CIN2/3(OR = 18.46,[9.33-36.51]). Nested case-control analysis observed a positive relationship between CD4,CD8 levels, and risk of CIN progression (CD4 OR = 0.34,[0.13, 0.94];CD8 OR = 0.27, [0.09, 0.79]),and an opposite relationship between IL-10 levels and risk of CIN progression (OR = 2.92, [1.09, 7.84]).

**Conclusions:**

Local vaginal CD4 and CD8 levels were positively correlated with CIN risk, and IL-10 levels were inversely correlated with CIN2/3, whether or not with high-risk HPV infection in Chinese women.

## 1 Introduction

Cervical intraepithelial neoplasia (CIN), now called cervical squamous intraepithelial (SIL), is a precancerous lesion of cervical cancer, reflecting the continuous process of cervical cancer occurrence and development. It is important to explore the risk factors of CIN to prevent cervical cancer (1, 2).High-risk HPV infection is a major cause of CIN (or SIL) and cervical cancer (3).The risk of CIN varies with the duration of high-risk HPV infection, immunosuppressive status, and other factors. The immune system may play an important regulatory role in the development of CIN and cervical cancer caused by high-risk HPV infection (4).Most women are able to clear the virus and diseased cells through their adaptive immune responses (5–7). But if the body’s immune function is disrupted or reduced, persistent high-risk HPV infection will significantly increase the risk of CIN and cervical cancer.

Cluster of differentiation (CD), also known as a leukocyte differentiation antigen, is a class of proteins or glycoproteins on cell membranes. CD molecules are often used as cell markers for immune antigen recognition, a technique that enables the observation of molecules on the cell surface and the identification of the cell. The most frequently used CD molecules are CD4 and CD8, which are commonly used as markers for helper T cells and cytotoxic T cells. It has been reported that the immune response after high-risk HPV infection relies mainly on two types of effector cells: CD4+ T cells (T helper cells, Th) and CD8+ T cells (cytotoxic T cells, CTL), which can clear the virus and diseased cells (8–12). Stimulated by local cytokines,CD4+ T cells differentiate into Th1 and Th2 cells.Th1 cells are mainly involved in cellular immunity through the secretion of IL-2, and Th2 cells are mainly involved in humoral immunity through IL-10 secretion (10). IL-2 is a vital cellular immune factor with antitumor effects (13). The team has elaborated on this in previous articles (14). Research on IL-10 is currently controversial. Some studies suggest that IL-10 is an immunosuppressive factor that promotes the occurrence of CIN or cervical cancer (15),while others consider it (11) an anti-tumor factor that inhibits the proliferation of cancer cells (16).IL-10 can increase the activation of CD8+ T cells (17).Persistent high-risk HPV infection means the immune system fails to clear the virus. HPV-induced cervical diseases, including CIN and cervical cancer, are linked to weak HPV-specific CD4+ and CD8+ T-cell responses (18).Impaired systemic or vaginal immunity can disrupt the balance of CD4+ and CD8+ T cells. Imbalances in CD4, CD8, the CD4/CD8 ratio, or IL-10 can impede the clearance of high-risk HPV, possibly leading to CIN. The effect and mechanism of action between the balance of immune cells and the changes in cytokine levels and high-risk HPV infection in the process of causing CIN and cervical cancer remain unclear, and there are few reports on this. Therefore, it is of great significance to explore the correlation between CD4, CD8, CD4/CD8, and IL-10 levels, and CIN risk, as well as whether there is a synergistic relationship with high-risk HPV infection, so as to block CIN progression in a timely manner.

The cervix is located in the vagina. Local vaginal immunity, as a crucial component of vaginal microecology, may play a more significant role than systemic immunity in preventing the development of cervical malignancies. The cervix is directly exposed to the vaginal environment, allowing both examinations and therapeutic interventions to be performed through a non-invasive route for direct targeting. Measuring the levels of local cytokines in the vagina may more accurately reflect its capacity to resist high-risk HPV infection (19). Few studies have investigated the impact of vaginal immunity on cervical lesions, so we chose vaginal lavage fluid to measure the levels of cytokines CD4, CD8, IL-10, and IL-2 (detailed in the team’s previous article),which can best represent the immune changes of the cervical environment and vaginal lavage fluid has higher sensitivity and specificity compared with blood and other body fluids.

We initiated a large population-based cervical cancer screening program and a prospective cohort study (Shanxi CIN Cohort) in Shanxi, China, in 2014-2015 (14, 20). The aim is to assess the potential risk factors related to CIN and to search for efficient strategies to prevent CIN from progressing to cervical cancer. In this study, we comprehensively evaluated the relevance of CD4, CD8, CD4/CD8, and IL-10 levels in local vaginal fluids in relation to the prevalence of CIN and the interactions between CD4, CD8, CD4/CD8, and IL-10 levels and high-risk HPV infection on CIN risk.

## 2 Material and methods

### 2.1 Study population

The data of this survey were sourced from the Shanxi CIN Cohort Study. In summary, free cervical cancer screenings were offered to eligible women with permanent residence in two counties(Yangqu and Jiexiu) of Shanxi Province, China, from 2014 to 2015. The study included 40,000 women aged 19 to 65 years old. All participants completed a questionnaire regarding their demographic characteristics and underwent a Pap test using liquid-based cytology (LBC). The participants with abnormal Pap test results were referred for colposcopy and histopathological examination. A total of 2,769 cases of atypical squamous cells of undetermined significance (ASC-US) and above were detected. With 10 cases of glandular cell abnormalities excluded and 68 cases of rejection, a total of 2,691 women underwent colposcopy and histopathological examination. Based on the pathological results, 19 women with cervical cancer were excluded, and 782 cases with CIN, including 564 cases of CIN grade 1 (CIN1), 171 cases of CIN grade 2(CIN2), and 47 cases of CIN grade 3(CIN3), were selected as the case group. There were 1,890 women with normal pathological results, 387 with incomplete data (they had not fully completed the three parts of the medical examination, including an in-person interview, physical examination, and clinical examination), and the remaining 1,503 women who voluntarily enrolled formed the control group. In the aggregate, 2,285 cases were included in the study. In addition, 1,503 cases in the control group were followed up. Among them, 25 cases with pathological results progressing to CIN (16 cases of CIN1, 6 cases of CIN2, and 3 cases of CIN3) were classified as the case group. According to a 1:3 ratio, 75 cases in the control group were matched based on the conditions of no lesion progression until the final follow-up time (January 31, 2019), age ± 1 year, and high-risk HPV infection status for the nested case-control study. The cohort was formed, consisting of 25 cases and 75 controls. We have already described in detail the rationale, design, and methodology of the study in our team’s previous article (14, 20–24). The study was approved by the Ethics Committee of the Second Hospital of Shanxi Medical University and registered in the Chinese Clinical Trial Register (ChiCTR) (registration number: ChiCTR-ROC-15006479).

### 2.2 Data collection

#### 2.2.1 Demographic characteristics and identifying factors related to cervical lesions

Our team (comprising gynecologists and epidemiologists) designed a closed questionnaire based on literature reviews, expert evaluations, and small-scale pilot surveys to assess the potential factors contributing to the occurrence of CIN. A face-to-face interview was conducted with the participants by trained and qualified researchers. The survey mainly covered demographic characteristics such as age, yearly income, alcohol drinking, smoking, and factors related to cervical lesions, parity, family history of cancer, and so on.

#### 2.2.2 Clinical data collection

Cervical exfoliated cells, HPV specimen collection, vaginal lavage fluid, and cervical tissue samples were collected following standard procedures. Pre-sampling: Participants avoided intravaginal medications or douching for 3 days, abstained from sex for 24 hours, and sampling was done outside menstruation. Cervical exfoliated cells collection: A speculum exposed the cervix. A cytobrush was used to collect cells from the transformation zone, rotated three times each way, and then placed in ThinPrep solution. The specimen was washed ten times, sealed, and stored at 4 °C for up to 7 days. Re-collecting the unsatisfactory cervical cell samples until satisfactory specimens are obtained. Unsatisfactory specimens in cervical cytology include inadequate cellularity and excessive obstructive factors. Inadequate cellularity is further classified as insufficient squamous epithelial cells or inadequate endocervical cell groups. For liquid-based cytology, a satisfactory sample must contain at least 500 well-preserved and morphologically clear squamous epithelial cells and at least 10 well-preserved endocervical cells or metaplastic cell groups. Samples falling below these thresholds are deemed unsatisfactory. Excessive obstructive factors refer to an overabundance of blood, inflammatory cells, coagulative necrotic debris, and similar obstructions in the specimen.

HPV specimen collection: Samples included cervical secretions and cervical exfoliated cells. A doctor used a speculum to insert an HPV brush into the cervical canal, rotated it five times, and placed it in a tube with preservation solution. The tube was labeled, sealed, and sent for testing. Samples were kept at 4 °C for up to 24 hours or -20 °C for up to 3 months. DNA extraction, PCR, and hybridization were performed.

Following the collection of HPV specimens, vaginal lavage was performed. Five milliliters of saline were used to rinse the upper third of the vagina and cervix, and the wash was collected, stored in a sterile tube, and used to measure CD4, CD8, and IL-10. The wash was centrifuged for 10 minutes at 2000 rpm, and the supernatant was aliquoted and stored at -80 °C (25).

Cervical tissue: According to the standard procedure (26), the suspicious cervical lesion tissue is removed using sterile biopsy forceps under colposcopy. The removed tissues are sent to the pathology department at once for pathological diagnosis.

#### 2.2.3 Clinical laboratory tests

##### 2.2.3.1 HPV DNA genotyping test, cervical pap cytology specimen test, and CD4 CD8 IL-10 test

Use the HPV GenoArray Test Kit to detect and genotype HPV. Based on the oncogenic potential, HPV testing was classified into negative, low-risk HPV, and high-risk HPV. In this study, six types of low-risk HPV (6, 11, 41, 42, 44, CP8304) and 15 types of high-risk HPV (16, 18, 31, 33, 35, 39, 45, 51, 52, 53, 56, 58, 59, 66, 68) can be detected.

An LBC method was used to perform all Pap tests. The film was prepared using a fully automatic liquid-based thin-layer cell production system, and fixed and Pap staining were performed in sequence. Two cytopathologists from our hospital read the films uniformly. If the pathological results are inconsistent, the senior cytopathologist will review them to ensure accuracy and consistency. The interpretation is carried out according to the Bethesda system classification method (TBS), revised in 2001.

According to the instructions for the enzyme-linked immunosorbent assay (ELISA) kits (Jinma Company in Shanghai), levels of CD4, CD8, and IL-10 were measured.

##### 2.2.3.2 Colposcopy and cervical histological examination

Colposcopy was performed by the colposcopy specialist at our hospital using an electronic colposcope (Shenzhen Goldway Company, China, model SLC2000). Gynecologists divide the cervix into four quadrants and examine each quadrant. A biopsy was performed in all areas that were visible to the naked eye and appeared abnormal. If no visible lesions were present, a random biopsy was performed at the junction of each quadrant scale column. Cytological results are abnormal, but colposcopy is negative or insufficient to perform endocervical curettage. Pathological sections were reviewed by two pathologists from our hospital, who were unaware of the cytology and HPV results. The diagnosis was then confirmed by senior pathologists. The cases were categorized as negative, CIN1, CIN2, CIN3, and squamous cell carcinoma.

### 2.3 Statistical analysis

We used EpiData 3.1 software to input data into two copies. The demographic characteristics and factors related to cervical lesions were described using descriptive statistics, including proportions, means, and standard deviations (SD). P-values for differences between groups were obtained from the chi-square test. Logistic regression models were used to determine the odds ratio (OR) and confidence interval (CI) of vaginal CD4, CD8, CD4/CD8, and IL-10 levels to the risk of CIN. The linear trends of the quartiles of CD4, CD8, CD4/CD8, and IL-10 levels were examined by using the quartile medians, which were treated as continuous variables. We further conducted a restricted cubic spline function analysis with three points (10th, 50th, and 90th percentiles) to evaluate the relationship between the levels of CD4, CD8, CD4/CD8, and IL-10 after logarithmic transformation, and CIN risk. The overall correlation between CD4, CD8, CD4/CD8, and IL-10 levels, and CIN risk after log conversion was evaluated using cubic splines to determine whether there is a nonlinear exposure-response relationship. We employed an interaction model to investigate the relationship between CD4, CD8, CD4/CD8, and IL-10 levels and high-risk HPV infection. The statistical analysis was performed using R software version 4.3.3. P < 0.05 was used as the standard to indicate that the difference is statistically significant.

## 3 Results

**Supplementary Table 1** presents the essential features of the excluded and included individuals with pathology-negative conditions. Apart from yearly income, characteristics such as age, education, alcohol drinking, and Marital status showed no statistically significant differences between the included and excluded women(P>0.05).

**Table 1** shows the characteristics of the 2285 women who received a cervical histologic examination. It is reported that women with CIN (including CIN1, CIN2, and CIN3) account for 34.2% of all women. There were statistically significant differences in indicators such as age, yearly income, alcohol drinking, family history of cancer, high-risk HPV infection, vaginal CD4, CD8,CD4/CD8, and IL-10 levels between the control group and the CIN group. The percentages of subjects aged >45 years were 64.3% in the CIN group and 68.7% in the control group (P value = 0.03). Individuals with a household yearly income ≥ 3000 yuan accounted for 23.1% of the CIN group and 19.2% of the control group (P value=0.03), and alcohol drinkers constituted 2.4% and 4.4% of each group (P value=0.02), respectively. Family history of cancer was reported in 13.3% of the CIN group compared to 8.8% of the control group (P value=0.01). The infection rates of high-risk HPV were 39.5% in the CIN group and 28.5% in the control group, respectively (P value<0.01). In the CIN group and the control groupe, the local vaginal CD4 levels were 8.67 (6.21-10.76) pg/ml and 7.26 (5.73-9.03) pg/ml (P value<0.01), CD8 levels were 6.07(4.50-8.03) pg/ml and 4.92 (3.66-6.51) pg/ml (P value<0.01), CD4/CD8 levels were 1.42 (1.05-1.89) and 1. 18 (1.55-1.88) (P value<0.01), and IL-10 levels were 65.79 (52.33-84. 11) pg/ml and 68.90 (53.70-86. 10) pg/ml (P value=0.04), respectively.

**Table 1:** Characteristics of 2,285 women with cervical histologic examination^1^ (%).

Characteristics	CIN		Total	*P value* ^2^
	No	Yes		
No. of participants	1503 (65.8)	782 (34.2)	2285 (100.0)	0.03
Age, years	49.4 ± 8.9	48.6 ± 9.2	49.2 ± 9.0	
≤ 45	470 (31.3)	279 (35.7)	749 (32.8)	
>45	1033 (68.7)	503 (64.3)	1536 (67.2)	
Education, years				
≤9	1295 (86.2)	675 (86.3)	1970 (86.2)	0.16
>9	208 (13.8)	107 (13.7)	315 (13.8)	
Yearly income, ¥				
<30000	1214 (80.8)	601 (76.9)	1815 (79.4)	0.03
≥30000	289 (19.2)	181 (23.1)	470 (20.6)	
Smoking				
No	1474 (98.1)	763 (97.6)	2237 (97.9)	0.43
Yes	29 (1.9)	19 (2.4)	48 (2.1)	
Alcohol drinking				
No	1437 (95.6)	763 (97.6)	2200 (96.3)	0.02
Yes	66 (4.4)	19 (2.4)	85 (3.7)	
Parity				
<3	1110 (73.9)	578 (73.9)	1688 (73.9)	0.98
≥3	393 (26.1)	204 (26.1)	597 (26.1)	
First sexual intercourse age, years				
<23	847 (56.4)	464 (59.3)	1311 (57.4)	0.17
≥23	656 (43.6)	318 (40.7)	974 (42.6)	
Family history of cancer				
No	1370 (91.2)	678 (86.7)	2048 (89.6)	0.01
Yes	133 (8.8)	104 (13.3)	237 (10.4)	
High-risk HPV infection				
Negative	1075 (71.5)	473 (60.5)	1548 (67.7)	<0.01
Positive	428 (28.5)	309 (39.5)	737 (32.3)	
CD4 (pg/ml)	7.26 (5.73-9.03)	8.67(6.21-10.76)	7.59 (5.88-9.78)	<0.01
CD8(pg/ml)	4.92 (3.66-6.51)	6.07(4.50-8.03)	5.24 (3.94-7.06)	<0.01
CD4/CD8	1.18(1.55-1.88)	1.42(1.05-1.89)	1.52 (1.12-1.89)	<0.01
IL-10 (pg/ml)	68.90(53.70-86.10)	65.79(52.33-84.11)	67.74(53.17-85.68)	0.04

^1^ Values are mean ± SD for normally distributed variables, median (25–75 percentiles) for skewed variables, or n (%) for categoric variables. CIN, cervical intraepithelial neoplasia.

^2^ P values for differences between groups were obtained from the chi-square test for categoric variables, and t-test for continuous variables.

**Figure 1:**
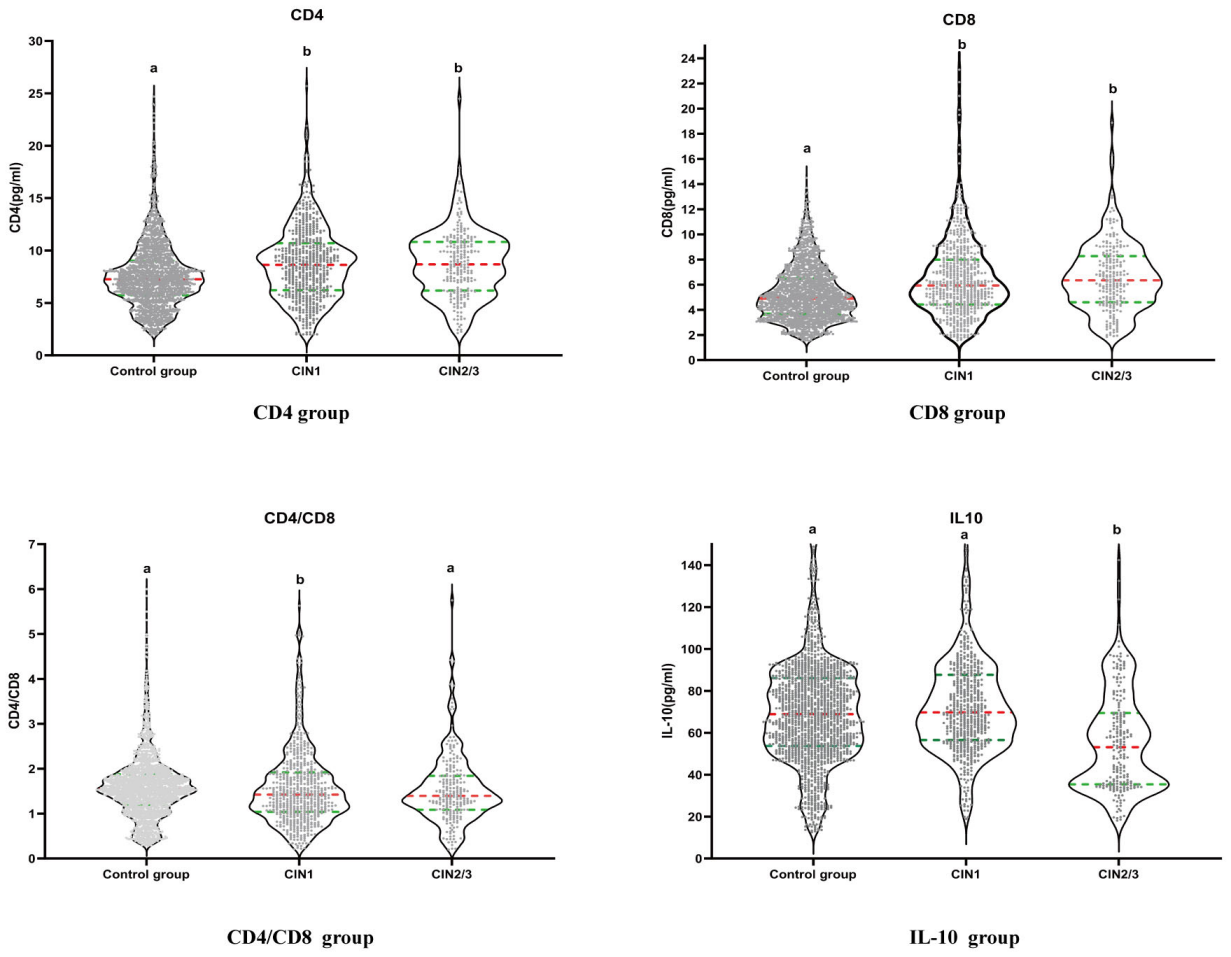
The distribution of CD4, CD8, CD4/CD8, and IL-10 levels in each group at baseline.

**Figure 1** shows the distribution of CD4, CD8, CD4/CD8 and IL-10 levels in the control group, CIN1 and CIN2/3 group. As **Figure 1** shows, the control group is labeled with the letter “a”. If there is no statistical difference in distribution between the control group and the CIN group, the CIN group will also be labeled as “a”. If there is a difference, it will be labeled as “b”. There were significant differences in the distribution of CD4 and CD8 between the control group (a) and the CIN1 group (b), as well as the CIN2/3 group (b). We observed a statistical difference in the distribution of CD4/CD8 between control group (a) and CIN1 group (b), but no statistical difference between control group (a) and CIN2/3 Group(a). There was a significant difference in the distribution of IL-10 between control group (a) and CIN2/3 group (b), but no significant difference between control group (a) and CIN1 group (a).

**Table 2** lists the correlations between the levels of CD4, CD8, CD4/CD8, IL-10, and high-risk HPV infection, as well as the occurrence of CIN in 2,285 participants. After adjusting for demographic characteristics, cervical lesions, and high-risk HPV infection fully, the levels of CD4 and CD8 in the vagina were positively associated with the prevalence of CIN 1 and CIN2/3.The 1st quartile levels of CD4 had the 55% and 68% decreased odds of CIN1 and CIN2/3 than the 4th quartile, respectively(for CIN1 OR = 0.45, 95% CI: 0.34, 0.60, P-trend < 0.01;for CIN2/3 OR = 0.32, 95% CI: 0.21, 0.48, P-trend < 0.01).The 1st quartile levels of CD8 had the 66% and 76% decreased odds of CIN1 and CIN2/3 than the 4th quartile, respectively(for CIN1 OR = 0.34, 95% CI: 0.26, 0.45, P-trend < 0.01;for CIN2/3 OR = 0.24, 95% CI: 0.16, 0.38, P-trend < 0.01).CD4/CD8 levels and the risk of CIN showed a trend of first decreasing and then increasing. When CD4/CD8 levels were between 1.53 and 1.89, the lowest prevalence of CIN1 and CIN2/3 was observed. The 3rd quartile levels of CD4/CD8 had the 39% and 30% decreased odds of CIN1 and CIN2/3 than the 4th quartile, respectively(for CIN1 OR = 0.61, 95% CI: 0.46, 0.82;for CIN2/3 OR = 0.70; 95% CI: 0.44, 1.11).There was no significant difference in the level of local IL-10 in the vagina and the occurrence of CIN1 (P-trend = 0.05), which was negatively correlated with the risk of CIN2/3. The 1st quartile levels of IL-10 had the 287% increased odds of CIN2/3 than the 4th quartile(OR = 3.87, 95% CI: 2.49, 6.00, P-trend < 0.01).The risk of developing CIN2/3 in women infected with high-risk HPV was 3.39 times higher than in women without high-risk HPV infection (OR = 4.39, 95% CI: 3.20-6.01).

**Table 2:** ORs and 95% CIs for the correlations between CD4 CD8 CD4/CD8 IL-10 levels and high-risk HPV infection with cervical intraepithelial neoplasia risk among 2,285 women^1^.

CIN	ORs (95% CIs)^2^		
	CIN	CIN1	CIN2/3
CD4(pg/ml)			
Q1 (≤ 5.88)	0.42 (0.33-0.54)	0.45 (0.34-0.60)	0.32 (0.21-0.48)
Q2 (5.89-7.59)	0.31 (0.24-0.41)	0.32 (0.24-0.43)	0.28 (0.18-0.43)
Q3 (7.60-9.78)	0.58 (0.45-0.73)	0.63 (0.48-0.82)	0.45 (0.30-0.67)
Q4 (≥ 9.79)	1.00 (Reference)	1.00 (Reference)	1.00 (Reference)
*P- trend*	< 0.01,1.15(1.11-1.19)	< 0.01,1.14(1.10-1.19)	< 0.01,1.19(1.13-1.26)
CD8(pg/ml)			
Q1 (≤ 3.94)	0.32 (0.25-0.41)	0.34 (0.26-0.45)	0.24 (0.16-0.38)
Q2 (3.95-5.24)	0.38 (0.30-0.49)	0.42 (0.32-0.55)	0.29 (0.19-0.45)
Q3 (5.25-7.06)	0.66 (0.52-0.84)	0.66 (0.51-0.86)	0.64 (0.44-0.94)
Q4 (≥ 7.07)	1.00 (Reference)	1.00 (Reference)	1.00 (Reference)
*P- trend*	< 0.01, 1.24(1.18-1.29)	< 0.01, 1.22(1.16-1.28)	< 0.01, 1.28(1.19-1.37)
CD4/CD8			
Q1 (≤ 1.12)	1.34 (1.05-1.71)	1.32 (1.01-1.73)	1.34 (0.88-2.05)
Q2 (1.13-1.52)	1.05 (0.82-1.34)	0.94 (0.71-1.24)	1.49 (0.99-2.25)
Q3 (1.53-1.89)	0.62 (0.48-0.81)	0.61 (0.46-0.82)	0.70 (0.44-1.11)
Q4 (≥ 1.89)	1.00 (Reference)	1.00 (Reference)	1.00 (Reference)
*P- trend*	P=0.003,0.78(0.67-0.92)	0.01,0.80(0.67-0.95)	0.03, 0.74(0.56-0.96)
IL-10(pg/ml)			
Q1 (≤ 53.17)	1.20 (0.93-1.54)	0.70 (0.52-0.94)	3.87 (2.49-6.00)
Q2 (53.18-67.74)	1.24 (0.97-1.59)	1.15 (0.88-1.51)	1.61 (0.99-2.63)
Q3 (67.75-85.68)	1.06 (0.83-1.37)	1.10 (0.84-1.43)	0.91 (0.53-1.57)
Q4 (≥ 85.69)	1.00 (Reference)	1.00 (Reference)	1.00 (Reference)
*P- trend*	0.09, 1.00(0.99-1.00)	0.05, 1.01(1.00-1.01)	< 0.01, 0.97(0.96-0.98)
High-risk HPV infection			
Positive	1.58 (1.31-1.90)	1.04 (0.83-1.29)	4.39 (3.20-6.01)
Negative	1.00 (Reference)	1.00 (Reference)	1.00 (Reference)

^1^Values are ORs (95% CIs) obtained from logistic regression analysis, using the highest intake group as the reference, unless otherwise indicated. CIN, cervical intraepithelial neoplasia.

^2^adjusted for age, educational level, yearly income, smoking, alcohol drinking, parity, first sexual intercourse age, and family history of cancer and high-risk HPV infection.

Among 2,285 women, 1,548 were HPV-negative, 15 were infected with low-risk HPV, and 722 were infected with high-risk HPV. The median values of CD4, CD8, CD4/CD8, and IL-10 in 1548 HPV-negative women were 7.53 pg/ml, 5.27 pg/ml, 1.52, and 68.47 pg/ml, respectively. The median of CD4,CD8,CD4/CD8,IL-10 in 15 women with low-risk HPV infection were 6.73pg/ml,5.98pg/ml,1.57and 73.39pg/ml, respectively. Among 722 women with high-risk HPV infections the median of CD4,CD8,CD4/CD8,IL-10 were 7.83pg/ml,5.16pg/ml,1.52and 66.44pg/ml, respectively(**Supplementary Table 2**).We observed a statistically significant difference between negative and low-risk HPV(P<0.01),negative and high-risk HPV patients of CD4,CD8,CD4/CD8, and IL-10(P<0.01). But a statistical difference for the four of them between high-risk HPV and low-risk HPV women was not observed(P>0.05).

**Figure 2:**
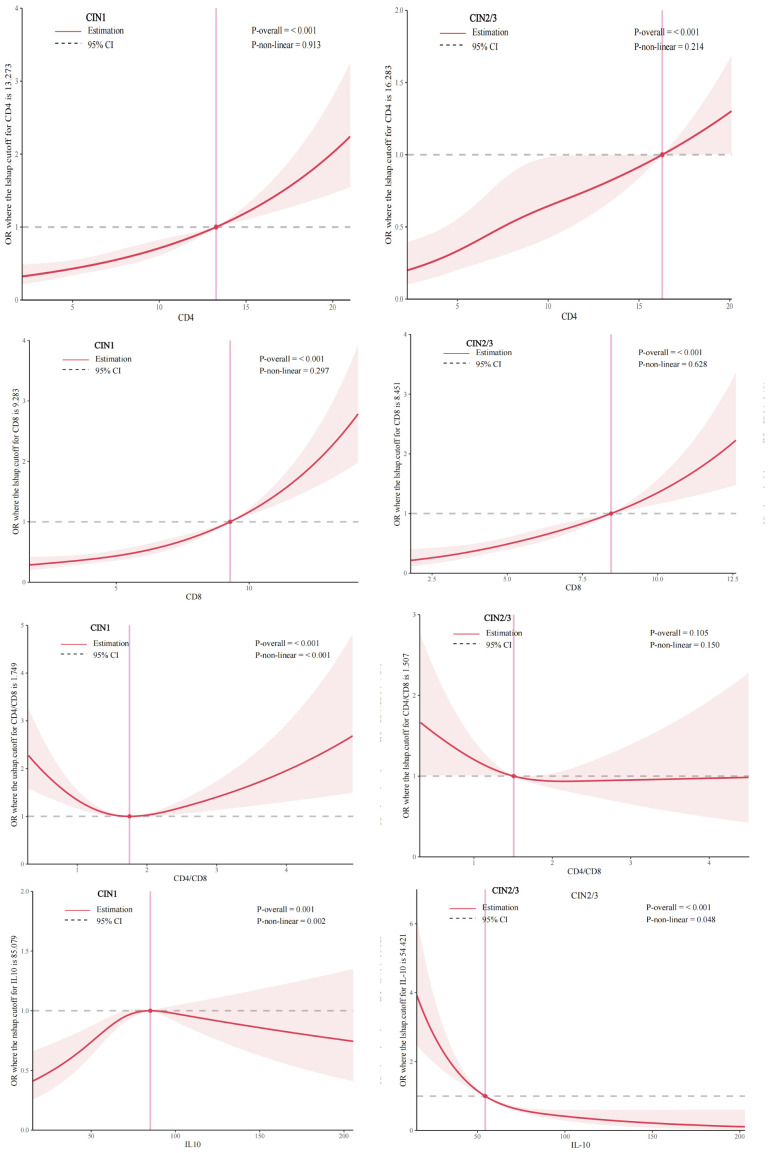
Dose-response relationship between CD4 CD8 CD4/CD8 IL-10 levels and prevalence of CIN 1, CIN 2/3 (restricted cubic spline models). The solid line represents the OR from the adjusted restricted cubic polynomial spline. The shaded area is the 95% CI. Adjusted for age, educational level, yearly income, smoking, alcohol drinking, parity, first sexual intercourse age, family history of cancer, and high-risk HPV infection. CIN, cervical intraepithelial neoplasia.

**Figure 2** shows after considering all potential factors including high-risk HPV infection, we observed the positive linear dose-response correlation between CD4,CD8 levels and CIN1, CIN2/3(CIN1:P_CD4_ overall < 0.01, P_CD4_ nonlinear = 0.91; P_CD8_ overall < 0.01, P_CD8_ nonlinear = 0.30; CIN2/3: P_CD4_ overall < 0.01, P_CD4_ nonlinear=0.21, P_CD8_ overall<0.01, P_CD8_ nonlinear= 0.63). The nonlinear relationship between CD4/CD8 levels and CIN1 was “U-shaped”, with a cut-off value of 1.75 (P_CD4/CD8_ overall<0.01, P_CD4/CD8_ nonlinear <0.01).We did not find the association between CD4/CD8 levels and CIN2/3 risk (P_CD4/CD8_ overall= 0.11, P_CD4/CD8_ nonlinear= 0.15). The nonlinear relationship between IL-10 level and CIN1 was “n-shaped”, with a cut-off value of 85.08 pg/ml (P_IL-10_ overall<0.01, P_IL-10_ nonlinear<0.01), while the inverse linear relationship between IL-10 level and CIN2/3 risk was observed (P_IL-10_ overall<0.01, P_IL-10_ nonlinear = 0.05).

**Figure 3:**
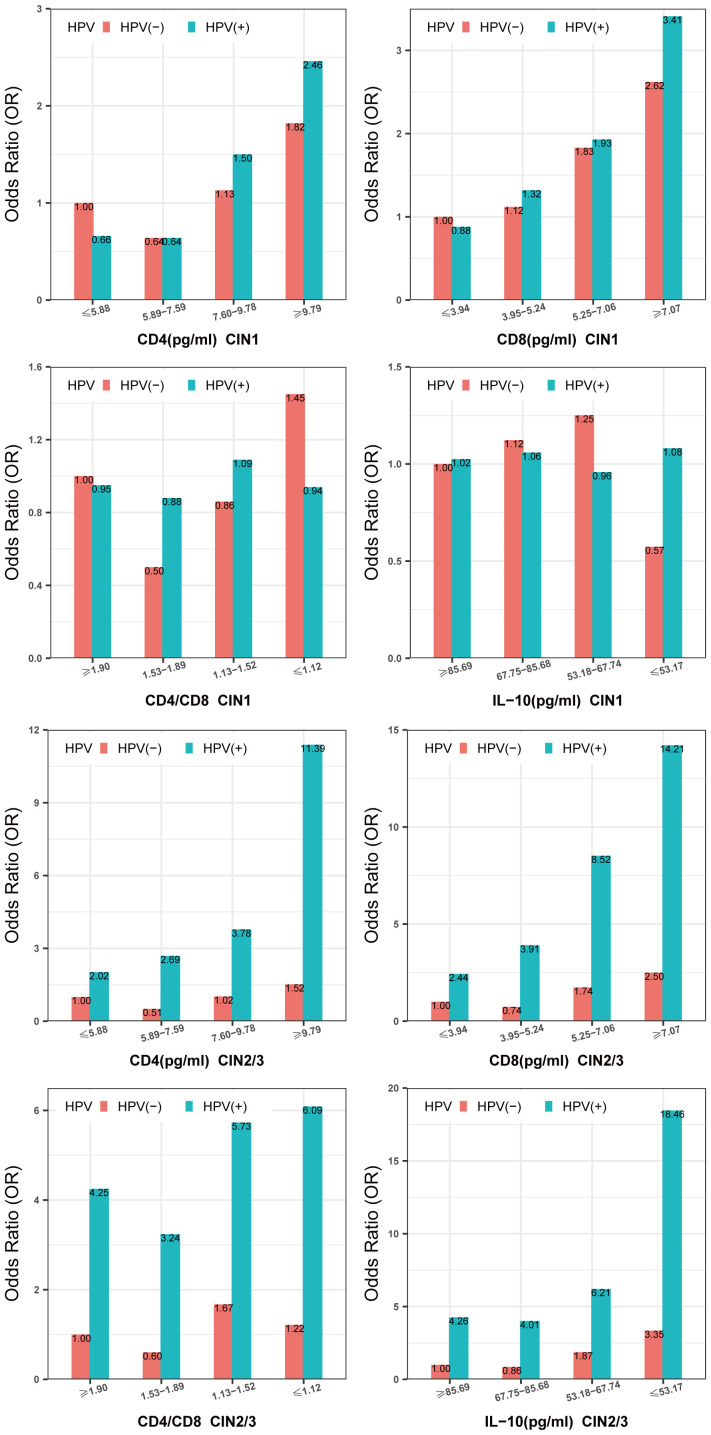
Multiplicative interaction between CD4,CD8,CD4/CD8,IL-10 level and high-risk HPV infection with prevalence of CIN. ORs are adjusted by age, educational level, yearly income, smoking, alcohol drinking, parity, first sexual intercourse age, family history of cancer. CIN, cervical intraepithelial neoplasia. HPV, high-risk HPV.

**Figure 3** and **Supplementary Table 3** show that the highest risk of CIN1 was observed in women with high-risk HPV infections, who also had the highest CD4 and CD8 levels (CD4≥9.79pg/ml; OR = 2.46, 95% CI: 1.61, 3.76;CD8≥7.07pg/ml, OR = 3.41, 95% CI: 2.20, 5.28). The lowest prevalence of CIN1 was observed when high-risk HPV was negative and CD4/CD8 levels were in the range of 1.53-1.89. There was no significant interaction between vaginal local IL-10 levels and high-risk HPV infection in patients with CIN1 (P_IL-10_ interaction=0.07). The highest risk of CIN2/3 was observed in women with high-risk HPV infections, who also had the highest CD4,CD8 levels (CD4≥9.79pg/ml; OR = 11.39, 95% CI: 6.45, 20.12;CD8≥7.07pg/ml, OR = 14.21, 95% CI: 7.53, 26.79) and the lowest CD4/CD8, IL-10 levels (CD4/CD8 ≤ 1.12; OR = 6.09, 95% CI: 3.25, 11.40;IL-10 ≤ 53.17pg/ml, OR = 18.46, 95% CI: 9.33, 36.51) (**Figure 3**; **Supplementary Table 4**). However, we did not find a significant difference between CD4, CD8, CD4/CD8, and IL-10 and high-risk HPV infection in our study (P > 0.05) (**Supplementary Table 5**).

We compared the levels of CD4, CD8, CD4/CD8, and IL-10 before and after the follow-up in the nested case-control survey. The median of CD4,CD8,CD4/CD8, and IL-10 levels before follow-up in the progression group (n=25) was 7.16 pg/ml, 5.63 pg/ml, 1.66, and 65.90 pg/ml. The median of CD4,CD8,CD4/CD8,IL-10 levels at end of follow-up was 8.98pg/ml, 7.23pg/ml,1.30 and 34.90pg/ml. The median levels of CD4, CD8, CD4/CD8, and IL-10 in the baseline data of non-progressive women (n = 75) were 7.75 pg/mL, 5.54 pg/mL, 1.53, and 69.70 pg/mL, respectively. The median levels of CD4, CD8, CD4/CD8, and IL-10 at the end of follow-up were 6.02 pg/ml, 5.51 pg/ml, 1.14, and 57.10 pg/ml, respectively.

After considering all potential confoundering factors, those with local vaginal CD4 ≤6.65 pg/ml (median) were 34% (OR = 0.34,95% CI: 0.13,0.94) of the risk of CIN in those with CD4>6.65 pg/ml; CD8 ≤ 6.05 pg/ml (median) was 27% (OR = 0.27,95% CI: 0.09,0.79) of those with CD8>6.05 pg/ml. We did not observe a significant association between CD4/CD8 ratios (P = 0.26) and the risk of CIN. Those with local vaginal IL-10 ≤ 55.90 pg/ml (median) were 2.92 times more likely to progress to CIN compared to those with IL-10 >55.90 pg/ml (OR = 2.92,95% CI: 1.09,7.84)(**Table 3**). However, we did not find a significant difference between CD4, CD8, CD4/CD8, and IL-10 and high-risk HPV infection in the follow-up (P > 0.05) (**Supplementary Table 6**).

**Table 3:** Logistic regression analysis of CD4, CD8, CD4/CD8, IL-10 levels and risk of CIN in follow-up (n=100).

	ORs (95% CIs)^1^	*P value*
CD4 (pg/ml)		
Q1 (≤ 6.65)	0.34 (0.13-0.94)	0.04
Q2 (>6.65)	1.00 (Reference)	
CD8 (pg/ml)		
Q1 (≤ 6.05)	0.27 (0.09-0.79)	0.02
Q2 (>6.05)	1.00 (Reference)	
CD4/CD8		
Q1 (≤ 1.26)	0.57 (0.22-1.50)	0.26
Q2 (>1.26)	1.00 (Reference)	
IL-10(pg/ml)		
Q1 (≤ 55.90)	2.92 (1.09-7.84)	0.03
Q2 (>55.90)	1.00 (Reference)	

## 4 Discussion

In this large-scale Shanxi CIN cohort study, CD4 and CD8 levels in the vagina were observed to be positively correlated with the risk of CIN1 and CIN2/3. There was a linear dose-response association. The non-linear relationship between IL-10 level and CIN1 was “n-shaped.” In contrast, there was an inverse linear relationship between IL-10 level and CIN2/3 risk. The nonlinear relationship between CD4/CD8 levels and CIN1 was “U-shaped.” We did not find an association between CD4/CD8 levels and the risk of CIN2/3 in the baseline population. Nor did we find a statistical association between CD4/CD8 levels and the risk of CIN in the follow-up population. So the associations between local CD4/CD8 levels in the vagina and the risk of CIN are not clear in our study. Women with the highest CD4 and CD8 levels and high-risk HPV infections in the vagina had the highest incidence rate of CIN1 and CIN2/3. Women with the lowest IL-10 levels and high-risk HPV infections had the highest incidence rate of CIN2/3. These results suggest the potential effects of CD4, CD8, and IL-10 levels in vaginal lavage in the occurrence of CIN2/3, with or without high-risk HPV infections.

The mucosal immunity of the female reproductive tract serves as the primary defense, coordinated by immune cells, pathogens, and cytokines. The presence of local immune cells and cytokines more accurately reflects the reproductive system’s immune function than systemic indicators, such as serum cytokines (27). Cervical cancer and CIN are linked to high-risk HPV infection, which can modify both systemic and local immunity (28, 29). Cell surface receptors exhibit a strong affinity for cytokines, and their interaction markedly enhances biological activity. Therefore, local immune cells and cytokines offer a more precise evaluation of local immunity than serum measurements (11, 19). Additionally, vaginal lavage fluid specimens are advantageous due to their non-invasive, readily accessible nature.

### 4.1 Association between CD4 CD8 levels and CIN risk

High-risk HPV infection is the primary cause of CIN and cervical cancer (3). However, the presence of high-risk HPV alone may not be sufficient to cause these diseases (8). An imbalance in the host immune response, along with certain cofactors, is also necessary to lead to cervical lesions. Thus, viral factors and host immune responses work together to determine if exposure to high-risk HPV will cause infection, whether the infection will continue, and if it will progress to CIN or cervical cancer (30).The immune response includes three aspects: immune clearance, immune balance, and immune escape. Both cellular and humoral immunity play a role in clearing viruses and diseased cells. Anti-tumor and antiviral effectors include the Th1 subset of CD4+ T cells (expressing cytokines IFN-γ and IL-2), the Th2 subset of CD4+ T cells (expressing cytokine IL-10), CD8+ T cells (clearing virus-infected and diseased cells), and natural killer (NK) cells (8).An effective immune response, including immune clearance and immune balance, can clear most high-risk HPV and lead to the regression of CIN-diseased cells (9).Persistent high-risk HPV infection of the cervix initiates cell transformation into new tumors. It can activate CD4+ T cells and CD8+ T cells, mediating an immune response that specifically targets cervical lesions and protects people from cervical cancer. Studies have found that in patients whose CIN regresses, the number of CD4+ T and CD8+ T cells increases. These cells fight against the viral oncoproteins E6 and E7 produced by high-risk HPV (31–33),thus exerting immune clearance. CD8+ T cells eliminate HPV-infected cervical epithelial cells and promote a sustained anti-tumor response, thereby preventing tumor growth (12).Dendritic cells presenting viral antigens can activate naive T cells. These then proliferate and differentiate into effector cells (CD4+ T cells, CD8+ T cells). Effector cells produce cytokines (IL-2, IFN-γ, IL-10), which amplify antiviral responses or specifically recognize and eliminate virus-infected cells (10).Studies show that as the grade of CIN increases, CD4+ and CD8+ T cell levels in the cervix also rise (12). Similarly, our research found an increase in CD4 and CD8 levels in CIN (including CIN1, CIN2/3). These may help fight against virus-infected and diseased cells to promote CIN regression.

Relatively low numbers of CD4+ T cells and CD8+ T cells have also been observed in all three types of CIN (34).Other studies have shown that as the grade of CIN increases, the CD4+ T cell level decreases, while the CD8+ T cell level increases (35).In contrast, our data showed that with the upgrading of CIN, the local vaginal levels of CD4+ T cells and CD8+ T cells also increased. While there was an interaction with high-risk HPV infection, no association between high-risk HPV infection and vaginal localized CD4+ T cell, CD8+ T cell, or CD4+/CD8+ T cell levels was observed. This discrepancy may be explained in several ways. First, with the increase in high-risk HPV infection and CIN lesions, the body initiates a specific cell-mediated immune response. Local immune cells, such as CD4+ T cells, CD8+ T cells, and dendritic cells, increase in number, actively playing a role in clearing high-risk HPV and diseased cells (11, 12).However, when high-risk HPV infection persists, high-risk HPV DNA integrates into the host cervical epithelial cells, leading to aberrant regulation of cell cycle control and secretion of E6 and E7 oncoproteins. These oncoproteins inhibit the immune system’s response to high-risk HPV and block activation of lymphocytes such as CD4+ T cells and CD8+ T cells, resulting in a dual performance of activation and inhibition, which can make the statistical results appear unrelated. Another explanation for the increase in CD4+ T cell and CD8+ T cell levels in CIN is that when immunosuppressive cells overwhelm immune clearance, immune evasion is favored. As a result, high-risk HPV infection persists, inducing the accumulation of ineffective CD4+ T cells and CD8+ T cells in CIN, so CIN persists or progresses (8, 36, 37).Our previous research (14) found that the decrease in local vaginal IL-2 levels increases the risk of CIN. The ineffective CD4+ T cells and CD8+ T cells in the vaginal local area increase but cannot normally express cytokines such as IL-2 and IL-10. This leads to a decline in the levels of IL-2, IL-10, and other cytokines, thereby promoting the occurrence and progression of CIN. Therefore, increasing the local effective CD4+ T cells and CD8+ T cells in the vagina may promote regression of CIN.

T lymphocyte subsets are the main effector cells of local cellular immunity in the cervix. They remain relatively stable under normal physiological conditions. CD4+ T cells and CD8+ T cells are both important players in T cell-mediated local anti-tumor immune responses. These cells can induce and restrict each other, regulate immune function, and maintain the stability of the local immune environment. CD4+ T lymphocytes play an auxiliary and inducing role in initiating immune responses. They primarily handle antigen transfer, but can also differentiate and produce antibodies (38).CD8+ T cells serve as both suppressor and cytotoxic T cells. They can both suppress and enhance the immune response (38).When the immune function of the body, or specifically the vagina, is low, the balance between CD4+ and CD8+ T cells is disrupted. As a result, high-risk HPV infection is difficult to eliminate and may persist, leading to the occurrence of CIN. An abnormal decrease in CD4/CD8 levels suggests T lymphocyte immune dysfunction or immunosuppression. This can lead to immune escape of tumor cells and promote the proliferation, differentiation, invasion, and metastasis of malignant tumor cells, worsening CIN lesions. As the severity of CIN lesions increases, the immune system becomes even more compromised, and the abnormality in the CD4/CD8 ratio becomes more pronounced. Previous studies (12, 35, 39, 40)have shown that CD4/CD8 levels are negatively correlated with the risk of CIN1 and CIN2/3.In our study, however, local vaginal CD4/CD8 levels in relation to the risk of CIN1 (P nonlinear < 0.01) and CIN2/3 (P nonlinear = 0.15) followed a “U” shape, first decreasing and then increasing. This may be because, in the first phase, a moderate increase in CD4/CD8 levels shows effective immune activation against HPV. Early and effective activation and proliferation of CD4+ T cells drive this phase (31). Although CD8+ T cells are also activated, their absolute numbers may lag behind or depend on help from CD4+ T cells. This phase represents a strong and coordinated adaptive immune response (31). In the second phase, the CD4/CD8 ratio continues to rise. Persistent high-risk HPV infection repeatedly stimulates the immune system. As a result, CD8+ T cells gradually become exhausted. Their function is severely impaired, so they cannot clear the virus and infected cells effectively (12, 41). At this stage, the high ratio reflects immune dysregulation and the exhaustion of CD8+ T cells, not immune activation.

The study found a significant interaction between vaginal CD4/CD8 levels and high-risk HPV infection, which increased CIN2/3 risk. In follow-up, CD4/CD8 levels showed no link to CIN risk. These conflicting results indicate the need for more research into T cell expression in disease progression. Since CIN grade is related to T lymphocyte subsets, detecting these subsets clinically may help assess lesion severity and guide treatment (42).

### 4.2 Association between IL-10 levels and CIN risk

Originally described as a product of Th2 cells, IL-10 is now recognized as being produced by nearly all immune cells. IL-10 production is induced after high-risk HPV infection, and elevated IL-10 levels have been found in cervical exudate in high-risk HPV patients (43).There is controversy surrounding IL-10’s multifaceted role, as it may both suppress and promote tumor initiation and progression (44). One view is that IL-10 exerts immunosuppressive effects by inhibiting factors such as IL-2 and interferon-γ (IFN-γ). Some studies report increased IL-10 expression in CIN or cervical cancer patients, supporting its association with the development of CIN and cervical cancer (13, 15, 45–47).Conversely, IL-10 is also reported to have anti-tumor effects, with immunostimulatory and anti-angiogenic properties that may inhibit tumor growth and spread, including that of cervical cancer cells (44, 48).It may promote NK cell proliferation and activity, aiding in the elimination of diseased cells. Several studies suggest that decreased IL-10 levels contribute to the development of CIN or malignancy (49–51). Our findings are consistent with studies showing that the risk of CIN2/3 increases as IL-10 levels in vaginal lavage fluid decrease. The highest risk of CIN2/3 was seen with high-risk HPV infection and the lowest IL-10 levels. Mechanistically, IL-10 primarily acts through the Janus kinase-signal transducer and activator of transcription (JAK-STAT) pathway; STAT1 is involved in the antiviral response. IL-10 induces the STAT1 pathway, increases granzyme and IFN-γ expression, enhances immune response, and promotes tumor cell apoptosis. Mouse studies indicate that high concentrations of pegylated IL-10 can induce complete tumor rejection.

The advantages and disadvantages have already been described in previous articles (14).

To summarize, this large-scale population study revealed clear relationships between immune markers and the risk of CIN. Higher local vaginal CD4 and CD8 levels were each independently associated with increased CIN risk. In contrast, lower IL-10 levels were associated with a greater risk of CIN2/3. Notably, when changes in these immune markers co-occurred with high-risk HPV infection, the risk of developing CIN or CIN2/3 increased more than would be expected from the effects of either factor alone, demonstrating a synergistic interaction between immune status and HPV infection.

Due to the accessibility of the cervix, measuring and adjusting local vaginal levels of CD4, CD8, and IL-10 is relatively convenient. Detecting and modulating these immune markers can complement cervical cancer screening, assist in regulating vaginal microecology to help prevent the progression of CIN and promote its regression (52). The specific meanings and measures include: 1. assessing local immune status; 2.predicting the ability to clear HPV; 3. aiding in triage by distinguishing between persistent and transient infections, which can identify cases requiring treatment and help reduce overtreatment (53); and 4.monitoring treatment response and prognosis, as the normalization of local cytokine levels after treatment may indicate a lower risk of recurrence. In the future, by locally administering therapeutic cervical cancer vaccines to induce local immune responses, the progression of CIN can be prevented and its regression promoted (53, 54).The effectiveness of such induction may be evaluated by monitoring changes in local vaginal CD4, CD8, and IL-10 levels.

## Data Availability

The original contributions presented in the study are included in the article/**Supplementary Material**. Further inquiries can be directed to the corresponding author.
