# Supplementary material for: A population-based study of interactions between high-risk human papillomavirus infection and vaginal local cytokines CD4 CD8 IL-10 with cervical intraepithelial neoplasia
Source: Front Oncol. 2025 Oct 10;15:1634489. doi: 10.3389/fonc.2025.1634489 (PMC12549257; doi:10.3389/fonc.2025.1634489)
Supplement: Supplementary file 1 [file Table1.docx]

Supplementary Material

# 1 Supplementary Table 1: The socio-demographic characteristics of included and exclude women with normal cervix

| **Characteristics** | **Women with normal cervix (n= 1890)** | | ***P value*** |
| --- | --- | --- | --- |
|  | **Included**  **(n= 1,503)** | **Excluded**  **(n= 387)** |  |
| **Age (years)** |  |  |  |
| < 35 | 114 (7.6) | 37 (9.6) | 0.14 |
| 35-44 | 317 (21.1) | 90 (23.3) |  |
| 45-54 | 571 (38.0) | 154 (39.8) |  |
| 55-64 | 495 (32.9) | 106 (27.4) |  |
| > 65 | 6 (0.4) | 0 (0.0) |  |
| **Education , years** |  |  |  |
| 0-6 | 306 (20.4) | 66 (17.1) | 0.25 |
| 7-9 | 633 (42.1) | 178 (46.0) |  |
| > 9 | 564 (37.5) | 143 (37.0) |  |
| **Yearly income, ¥** |  |  |  |
| <10000 | 173 (11.5) | 18 (4.7) | <0.01 |
| 10000-30000 | 738 (49.1) | 137 (35.4) |  |
| >30000 | 592 (39.4) | 232 (59.9) |  |
| **Smoking** |  |  |  |
| No | 1474 (98.1) | 378 (97.7) | 0.62 |
| Yes | 29 (1.9) | 9 (2.3) |  |
| **Marital status** |  |  |  |
| Married | 1408 (93.7) | 368 (95.1) | 0.30 |
| Others | 95 (6.3) | 19 (4.9) |  |

**Supplementary Table 2:**The median values of CD4, CD8, CD4/CD8, and IL-10 levels grouped by HPV infection in 2,285 women

| **Cytokines** | HPV-negative**（n=1548）** | Low-risk HPV infection**（**n=15**）** | High-risk HPV infection**（**n=722**）** |
| --- | --- | --- | --- |
| **CD4 (pg/ml)** | 7.53 | 6.73 | 7.83 |
| **CD8 (pg/ml)** | 5.27 | 5.98 | 5.16 |
| **CD4/CD8** | 1.52 | 1.57 | 1.52 |
| **IL-10(pg/ml)** | 68.47 | 73.39 | 66.44 |

**Supplementary Table 3:**Interaction between CD4, CD8, CD4/CD8, IL-10 levels and high-risk HPV infection on the risk of CIN1 in 2,285 women

| **CIN1** | **HPV** | **ORs (95% CIs)^1^** | ***P value*** |
| --- | --- | --- | --- |
| **CD4 (pg/ml)** |  |  |  |
| ≤5.88 | HPV（-） | 1.00 (Reference) |  |
|  | HPV（+） | 0.66 (0.41-1.07) | 0.09 |
| 5.89-7.59 | HPV（-） | 0.64 (0.45-0.91) | 0.01 |
|  | HPV（+） | 0.64 (0.40-1.04) | 0.07 |
| 7.60-9.78 | HPV（-） | 1.13 (0.81-1.58) | 0.46 |
|  | HPV（+） | 1.50 (1.00-2.25) | 0.05 |
| ≥9.79 | HPV（-） | 1.82(1.32-2.51) | <0.01 |
|  | HPV（+） | 2.46(1.61-3.76) | <0.01 |
| **CD8 (pg/ml)** |  |  |  |
| ≤3.94 | HPV（-） | 1.00 (Reference) |  |
|  | HPV（+） | 0.88 (0.55-1.42) | 0.60 |
| 3.95-5.24 | HPV（-） | 1.12 (0.78-1.61) | 0.53 |
|  | HPV（+） | 1.32 (0.84-2.06) | 0.23 |
| 5.25-7.06 | HPV（-） | 1.83 (1.29-2.57) | <0.01 |
|  | HPV（+） | 1.93 (1.23-3.03) | <0.01 |
| ≥7.07 | HPV（-） | 2.62 (1.87-3.67) | <0.01 |
|  | HPV（+） | 3.41 (2.20-5.28) | <0.01 |
| **CD4/CD8** |  |  |  |
| ≥1.90 | HPV（-） | 1.00 (Reference) |  |
|  | HPV（+） | 0.95 (0.62-1.45) | 0.80 |
| 1.53-1.89 | HPV（-） | 0.50 (0.35-0.71) | <0.01 |
|  | HPV（+） | 0.88 (0.58-1.35) | 0.56 |
| 1.13-1.52 | HPV（-） | 0.86 (0.62-1.19) | 0.36 |
|  | HPV（+） | 1.09 (0.72-1.66) | 0.68 |
| ≤1.12 | HPV（-） | 1.45 (1.06-1.98) | 0.02 |
|  | HPV（+） | 0.94 (0.60-1.47) | 0.78 |
| **IL-10 (pg/ml)** |  |  |  |
| ≥85.69 | HPV（-） | 1.00 (Reference) | 0.01 |
|  | HPV（+） | 1.03 (0.67-1.56) | 0.91 |
| 67.75-85.68 | HPV（-） | 1.12 (0.82-1.54) | 0.48 |
|  | HPV（+） | 1.06 (0.70-1.61) | 0.79 |
| 53.18-67.74 | HPV（-） | 1.25 (0.91-1.72) | 0.17 |
|  | HPV（+） | 0.96 (0.62-1.47) | 0.85 |
| ≤53.17 | HPV（-） | 0.57 (0.40-0.83) | 0.00 |
|  | HPV（+） | 1.08 (0.69-1.69) | 0.73 |

^1^Adjusted for age, educational, yearly income, smoking, alcohol drinking, parity, first sexual intercourse age and family history of cancer

**Supplementary Table 4:**Interaction between CD4, CD8, CD4/CD8, IL-10 levels and highrisk HPV infection on the risk of CIN2/3 in 2,285 women

| **CIN2/3** | **HPV** | **ORs (95% CIs)**1 | ***P value*** |
| --- | --- | --- | --- |
| **CD4** (pg/ml) |  |  |  |
| ≤5.88 | HPV（-） | 1.00 (Reference) |  |
|  | HPV（+） | 2.02 (1.05-3.87) | 0.04 |
| 5.89-7.59 | HPV（-） | 0.51 (0.24-1.05) | 0.07 |
|  | HPV（+） | 2.69 (1.46-4.98) | 0.00 |
| 7.60-9.78 | HPV（-） | 1.02 (0.54-1.96) | 0.94 |
|  | HPV（+） | 3.78 (2.07-6.91) | 0.00 |
| ≥9.79 | HPV（-） | 1.52 (0.82-2.82) | 0.19 |
|  | HPV（+） | 11.39 (6.45-20.12) | 0.00 |
| **CD8 (pg/ml)** |  |  |  |
| ≤3.94 | HPV（-） | 1.00 (Reference) |  |
|  | HPV（+） | 2.44 (1.20-4.98) | 0.01 |
| 3.95-5.24 | HPV（-） | 0.74 (0.33-1.65) | 0.46 |
|  | HPV（+） | 3.91 (1.99-7.66) | 0.00 |
| 5.25-7.06 | HPV（-） | 1.74 (0.88-3.43) | 0.11 |
|  | HPV（+） | 8.52 (4.48-16.81) | 0.00 |
| ≥7.07 | HPV（-） | 2.50 (1.30-4.81) | 0.01 |
|  | HPV（+） | 14.21 (7.53-26.79) | 0.00 |
| **CD4/CD8** |  |  |  |
| ≥1.90 | HPV（-） | 1.00 (Reference) |  |
|  | HPV（+） | 4.25 (2.23-8.09) | 0.00 |
| 1.53-1.89 | HPV（-） | 0.60 (0.28-1.30) | 0.20 |
|  | HPV（+） | 3.24 (1.66-6.31) | 0.00 |
| 1.13-1.52 | HPV（-） | 1.67 (0.89-3.61) | 0.11 |
|  | HPV（+） | 5.73 (3.06-10.73) | 0.00 |
| ≤1.12 | HPV（-） | 1.22 (0.61-2.43) | 0.58 |
|  | HPV（+） | 6.09 (3.25-11.40) | 0.00 |
| **IL-10 (pg/ml)** |  |  |  |
| ≥85.69 | HPV（-） | 1.00 (Reference) |  |
|  | HPV（+） | 4.26 (1.98-9.17) | 0.00 |
| 67.75-85.68 | HPV（-） | 0.86 (0.35-2.12) | 0.74 |
|  | HPV（+） | 4.01 (1.84-8.75) | 0.00 |
| 53.18-67.74 | HPV（-） | 1.87 (0.87-4.01) | 0.11 |
|  | HPV（+） | 6.21 (2.98-12.91) | 0.00 |
| ≤53.17 | HPV（-） | 3.35 (1.67-6.72) | 0.00 |
|  | HPV（+） | 18.46 (9.33-36.51) | 0.00 |

^1^Adjusted for age, educational, yearly income, smoking, alcohol drinking, parity, first sexual intercourse age and family history of cancer

**Supplementary Table 5:** Logistic regression analysis of risk of CD4,CD8,CD4/CD8,IL-10 and high-risk HPV in baseline population

|  | **ORs (95% CIs)^1^** | ***P value*** |
| --- | --- | --- |
| **CD4 (pg/ml)** |  |  |
| Q1 (≤7.59) | 1.19 (0.97-1.47) | 0.10 |
| Q2 ( >7.59) | 1.00 (Reference) |  |
| **CD8 (pg/ml)** |  |  |
| Q1 (≤6.05) | 0.84 (0.67-1.06) | 1.52 |
| Q2 ( >6.05) | 1.00 (Reference) |  |
| **CD4/CD8** |  |  |
| Q1 (≤1.26) | 0.95 (0.76-1.19) | 0.66 |
| Q2 ( >1.26) | 1.00 (Reference) |  |
| **IL-10(pg/ml)** |  |  |
| Q1 (≤55.90) | 0.94 (0.78-1.12) | 0.48 |
| Q2 ( >55.90) | 1.00 (Reference) |  |

^1^Adjusted for age, educational, yearly income, smoking, alcohol drinking, parity, first sexual intercourse age and family history of cancer

**Supplementary Table 6:** Logistic regression analysis of risk of CD4,CD8,CD4/CD8,IL-10 and high-risk HPV in follow-up population(n=100)

|  | **ORs (95% CIs)^1^** | ***P value*** |
| --- | --- | --- |
| **CD4 (pg/ml)** |  |  |
| Q1 (≤6.65) | 0.52 (0.22-1.23) | 0.14 |
| Q2 ( >6.65) | 1.00 (Reference) |  |
| **CD8 (pg/ml)** |  |  |
| Q1 (≤6.05) | 0.79 (0.33-1.94) | 0.61 |
| Q2 ( >6.05) | 1.00 (Reference) |  |
| **CD4/CD8** |  |  |
| Q1 (≤1.26) | 0.81 (0.34-1.90) | 0.63 |
| Q2 ( >1.26) | 1.00 (Reference) |  |
| **IL-10(pg/ml)** |  |  |
| Q1 (≤55.90) | 1.38 (0.59-3.22) | 0.45 |
| Q2 ( >55.90) | 1.00 (Reference) |  |

^1^Adjusted for age, educational, yearly income, smoking, alcohol drinking, parity, first sexual intercourse age and family history of cancer

**2 Supplementary Figure1**


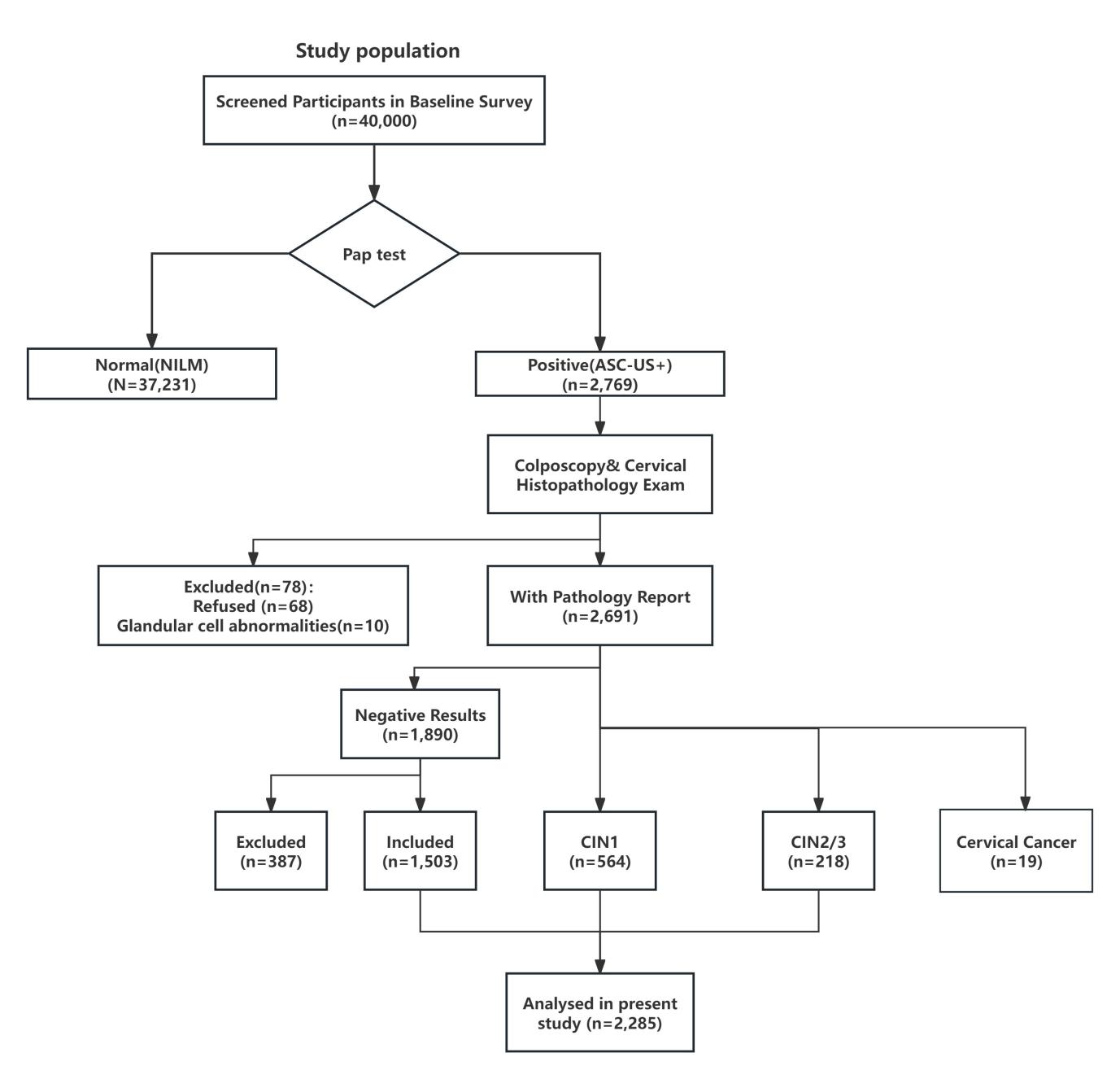


**Supplementary Figure1**

Flow diagram of participants in the study. ASC-US, atypical squamous cells of undetermined significance; CIN, cervical intraepithelial neoplasia;NILM, negative for intraepithelial lesion or malignancy

**Supplementary Figure 2**


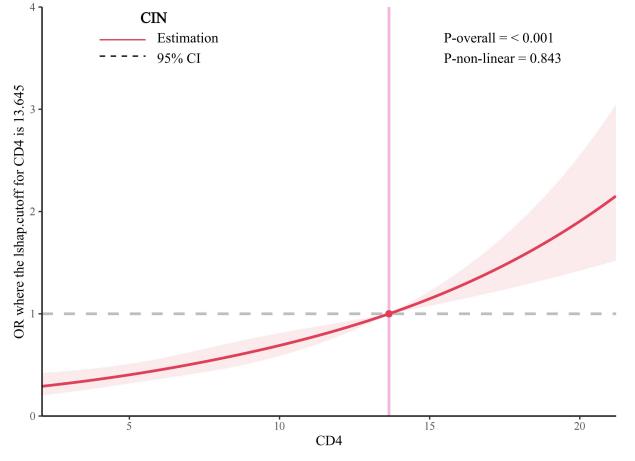

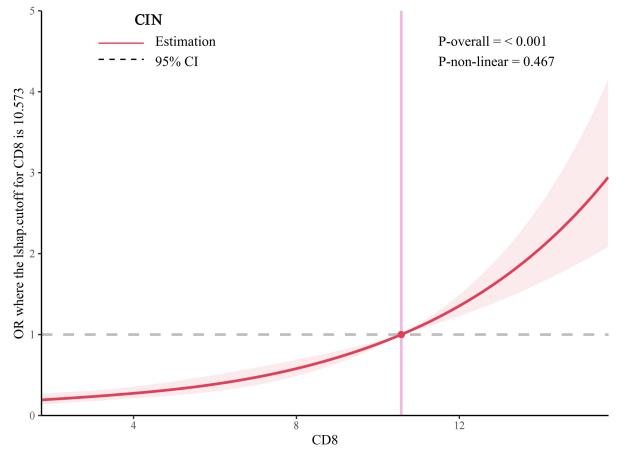


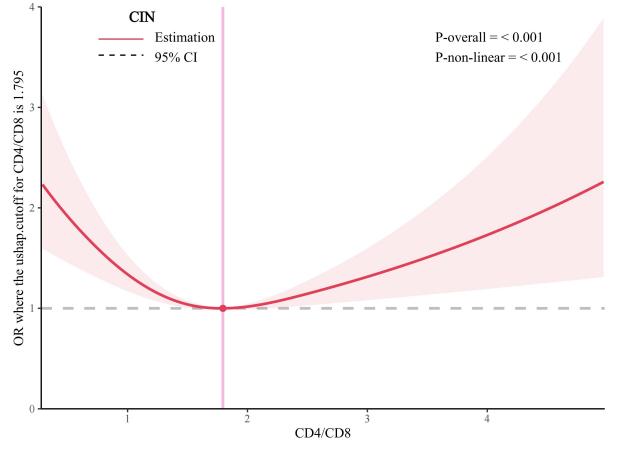

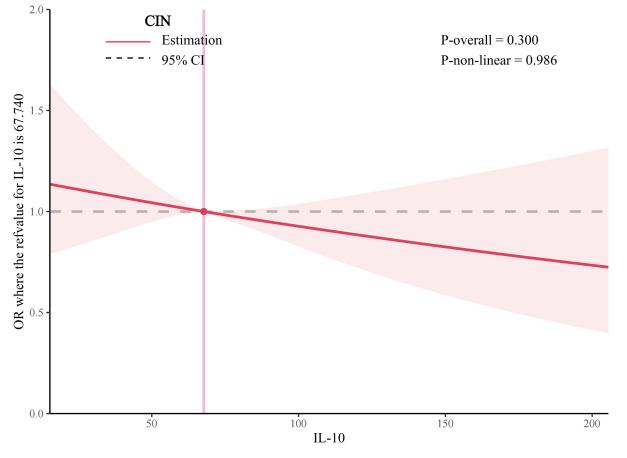


**Supplementary Figure 2:** Dose-response relationship between CD4 CD8 CD4/CD8 IL-10 levels with prevalence of CIN (restricted cubic spline models). The solid line represents the OR from the adjusted restricted cubic polynomial spline. The shaded area is the 95% CI. Adjusted for age, educational level, yearly income, smoking,

alcohol drinking, parity, first sexual intercourse age, family history of cancer, and high-risk HPV infection. CIN, cervical intraepithelial neoplasia.
